# Influence of Geometric Changes in the Thoracic Aorta due to Arterial Switch Operations on the Wall Shear Stress Distribution

**DOI:** 10.2174/1874120701711010009

**Published:** 2017-02-16

**Authors:** Tomohiro Fukui, Hiroaki Asama, Manabu Kimura, Toshiyuki Itoi, Koji Morinishi

**Affiliations:** 1 Department of Mechanical Engineering, Kyoto Institute of Technology, Matsugasaki Goshokaido-cho, Sakyo-ku, Kyoto 606-8585, Japan; 2 Kyoto Second Red Cross Hospital, Haruobi-cho 355-5, Kamigyo-ku, Kyoto 602-8026, Japan; 3 Department of Pediatric Cardiology and Nephrology, Graduate School of Medical Science, Kyoto Prefectural University of Medicine, Kajii-cho 465, Kamigyo-ku, Kyoto 602-0841, Japan

**Keywords:** Aneurysm, Arterial switch operation, Blood flow simulation, Curvature, Torsion, Transposition of the great arteries, Wall shear stress

## Abstract

**Background:**

The transposition of the great arteries (TGA) is one of the most severe congenital heart diseases. The arterial switch operation (ASO) is the preferred procedure to treat TGA. Although numerous reports have shown good results after ASOs, some patients suffer from circulatory system problems following the procedure. One reason for problems post-ASO is the local changes in the curvature and torsion of the thoracic aorta.

**Objective:**

The influence of these geometric changes on the blood flow field needs to be investigated in detail to consider possible cardiovascular problems after an ASO.

**Method:**

In this study, we conduct blood flow simulations in the thoracic aorta post-ASO, evaluate geometric changes in the aorta due to the ASO in terms of curvature and torsion, and consider the effect of geometric changes on blood flow in the aorta.

**Results:**

It was found that a large curvature near the aortic root causes an increase in the maximal wall shear stress value in the middle systole. Moreover, a large torsion results in a circumferential change in the maximal wall shear stress region. It was also found that the maximal wall shear stress in the post-ASO models is significantly higher than that in the normal models. This indicates that the aortic aneurysm initiation risk for a post-ASO artery may be higher than that of a normal artery.

**Conclusion:**

To reduce the risk of initiating an aneurism, it is suggested that the curvature near the aortic root should be decreased during the ASO.

## INTRODUCTION

The geometry of the thoracic aorta is complicated with varying curvature, torsion, branches, and tapering, and the area is well known for its localization of aortic aneurysms, particularly from the root of the ascending aorta to the aortic arch. This is attributed to the local wall shear stress distribution changes resulting from its complicated geometry. When the wall shear stress is locally high, the endothelial cells in the affected area produce vasodilators such as nitric oxide (NO). Then, the smooth muscle cells in the media relax, and the artery becomes dilated [1, 2]. When the arterial wall is exposed to long-term high shear stress, remodeling of the arterial tissues occurs, and this may initiate an aortic aneurysm [3-6]. Therefore, there is a causal relationship between high wall shear stress regions and the localization of aortic aneurysms from the point of view of rheology.

Kondo *et al.* [7] examined induced cerebral aneurysms arising at nonbranching sites in rats, and discussed the relationship between development of the aneurysms and curvatures of the vessels. They concluded that the site of origin is strongly related to hemodynamic stress. Shahcheraghi *et al.* [8] conducted a three-dimensional and pulsatile blood flow simulation in a human aortic arch and its three major branches, and observed an extensive secondary flow motion in the aorta, which was influenced considerably by the presence of the branches. They also considered the higher wall shear stress regions within the aorta and branches. Mori and Yamaguchi [9] demonstrated the relationship between the maximal wall shear stress position and the localization of aortic aneurysms using thoracic artery models with different torsions. They also showed that the maximal wall shear stress in an artery with an aneurysm is higher than that in a normal model. Nakamura *et al.* [10] and Pasta *et al.* [11] demonstrated the effects of the inlet condition on the wall shear stress distribution from the root of the aorta to the aortic arch using thoracic aortic models with left ventricle or aortic valves.

We focus on the transposition of the great arteries (TGA) model in this study. The TGA is one of the most severe congenital heart diseases. In normal circulation, blood from the left ventricle flows to the systemic circulation, and the pulmonary circulation starts from the right atrium and ventricle. In contrast, in TGA circulation, because the aorta arises from the right ventricle, blood in the systemic circulation is always rich in carbon dioxide and poor in oxygen. The systemic and pulmonary circulations are completely separated in TGA. The arterial switch operation (ASO) is the preferred procedure to treat TGA. Although numerous reports have shown good results after ASOs [12-14], some patients suffer from circulatory system problems following the procedure [15, 16]. One reason for problems post-ASO is the local changes in the curvature and torsion of the thoracic aorta [17]. The influence of these geometric changes on the blood flow field needs to be investigated in detail to consider possible cardiovascular problems after an ASO.

In this study, we conduct blood flow simulations in the thoracic aorta post-ASO, evaluate geometric changes in the aorta due to the ASO in terms of curvature and torsion, and consider the effect of geometric changes on blood flow in the aorta. We also conduct a wall shear stress analysis to assess the initiation and localization of an aortic aneurysm post-ASO.

## METHOD

### Computational Models

Three aortic models, including one normal and two post-ASO models, were prepared [17]. These three models were obtained from three individual patients. One is from a person with no disability in the aorta [9] for comparison, and the other two are from patients after ASOs. A schematic view of the three models, normal model A and post-ASO models B and C, are shown in Fig. (**1**). The numbers in the models indicate their representative cross section numbers, which correspond to the selected point numbers from the medical imaging data. Briefly, these artery models were constructed from the medical imaging data of the end diastole and the selected point number 12. The selected points were then smoothly interpolated by a cubic spline interpolation method to decide the centerline of each model. These models are assumed to be rigid, and have no branches, tapering, or aortic valves for simplicity.

Since the centerline of each model was expressed by the cubic spline curves in this study, the curvature *κ* and torsion *τ* along the centerline are simply obtained by the Frenet-Serret formulas as follows,

(1) $$\kappa =∥x^{''}∥$$

(2) $$\kappa^{2}\tau =(x^{'},x^{''},x^{'''})$$

where **x** is the position vector of the centerlines. The geometric shapes of the models are then compared in terms of their curvature and torsion in Fig. (**2**). Note that these values along the centerlines are not strongly dependent on the choice of the selected point numbers from the medical imaging data. In the normal model A, large curvature can be seen near the aortic root and arch. Conversely, in the post-ASO models, large curvature can be seen throughout the aortic root. This is a distinctive characteristic of the post-ASO models. The torsion of the normal model A is relatively flat, whereas that of the post-ASO models is not flat but rather varies over a wide range of the aorta.

### Governing Equations

The governing equations are the incompressible Navier-Stokes equations as shown in Eq. (3), where **q** = (*p*, *u*, *v*, *w*)^T^, **h** = (0, *u*, *v*, *w*)^T^, **E***_x_* = (*βu*, *u*^2^ + *p*, *uv*, *uw*)^T^, **E***_y_* = (*βv*, *vu*, *v*^2^ + *p*, *vw*)^T^, **E***_z_* = (*βw*, *wu*, *wv*, *w*^2^ + *p*)^T^, **E***_xv_* = (0, ∂*u*/∂*x*, ∂*v*/∂*x*, ∂*w*/∂*x*)^T^, **E***_yv_* = (0, ∂*u*/∂*y*, ∂*v*/∂*y*, ∂*w*/∂*y*)^T^, **E***_zv_* = (0, ∂*u*/∂*z*, ∂*v*/∂*z*, ∂*w*/∂*z*)^T^, *t’* is the pseudo-time, *t* is the physical time, and *β* is the artificial compressibility coefficient. All parameters in Eq. (3) are expressed by dimensionless values, then, dimensionless pressure *p*, for example, is defined as, $p=\tilde{p}/(\tilde{\rho }{\tilde{U}}^{2})$ where parameters with ~ denote physical quantities. Each term is solved as follows: the Lower-Upper Symmetric-Gauss-Seidel method was used for the pseudo-time term, the first-ordered backward difference method was used for the physical time term, Roe’s Flux Difference Splitting scheme and third-ordered weighted essentially non-oscillatory (ENO) method were used for the convective term, and the second-ordered central difference method was used for the viscous term. The artificial compressibility coefficient *β* was set to 1.0 in this study. Note that this artificial compressibility coefficient *β* doesn’t affect the final results [18], but only affects the number of time steps to converge in solving these governing equations.

(3) $$\frac{\partial q}{\partial t^{'}}+\frac{\partial h}{\partial t}+\frac{\partial E_{x}}{\partial x}+\frac{\partial E_{y}}{\partial y}+\frac{\partial E_{z}}{\partial z}=\frac{1}{R_{e}}\left(\frac{\partial E_{\mathrm{xv}}}{\partial x}+\frac{\partial E_{\mathrm{yv}}}{\partial y}+\frac{\partial E_{\mathrm{zv}}}{\partial z}\right)$$

### Virtual Flux Method

The arterial walls were expressed using the virtual flux method [19-21]. Briefly, the virtual flux method enables us to describe a curved boundary on a Cartesian grid in the same way as a famous immersed boundary method dose. The major difference between them is adding an external force or not. In the virtual flux method, fluid dynamic conditions on the body surface are considered, for example to satisfy the no-slip condition on the boundary, zero velocity and zero pressure gradient are considered, instead of adding external forces. A flux near the body surface in Eq. (3) is then replaced by a virtual flux, which satisfies the fluid dynamic conditions, to calculate a flow field with a curved boundary. In addition, good scalability for the parallelized computation is also expected due to the method’s simple implementation. See references above for more details.

### Computational Conditions

The computational properties are as follows. The diameter of the aorta was set to 20 mm and included 60 grids, which indicates spatial resolution $\Delta_{x}=\Delta_{y}=\Delta_{z}$ was 1/3 mm, and the number of grids was some 8 millions in total. The maximal velocity, *U*, and the aortic pressure, *p*, were 1.2 m/s and 100 mmHg, respectively. The viscosity was assumed to be 2.4 x 10^-5^ m^2^/s, accordingly, Reynolds number at the peak corresponded to 1000. The inlet velocity with the heart rate of 60 bpm is as shown in Fig. (**3**). The boundary conditions were no-slip on the wall, Poiseuille flow perpendicular to the cross section at the inlet, and constant aortic pressure at the outlet. The other parameters at the inlet and outlet were linearly extrapolated. The time step 
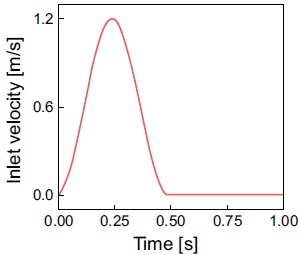
*t* was set to 1.0 x 10^-3^ s, and the blood flow simulation was carried out for four cardiac cycles to confirm the flow periodicity. Since a Cartesian rectangular parallelepiped computational domain was applied in our computation, there were many unnecessary grids to cover the aortic model. In order to reduce the total number of grids, five rectangular parallelepiped computational regions, which were connected with each other, were used, and a parallel computing using OpenMP was conducted.

## RESULTS

The flow fields at the cross section of point 4, which corresponds to the top of the aortic arch, are compared for the three models in Fig. (**4**), which shows the axial velocity contours and secondary flow vectors. A large pink arrow indicates the decentering eccentric direction of the velocity profile. In the early systole, the maximal axial velocity is decentered towards the inner wall side, while, in the middle systole, it is decentered towards the outer wall side. The secondary flow is mostly clockwise; however, it differs between the models. One large clockwise flow and one small counterclockwise secondary flow are observed in model A. Conversely, one large clockwise secondary flow can be seen in models B and C. To examine this decentered velocity profile, the curvature vectors at cross sections 3 and 4 are shown in Fig. (**5**). The curvature vectors at the cross sections are shown in black and the opposing vectors (in pink) are the centrifugal force vectors. In the early systole, the axial velocity is not sufficiently high and the velocity profile is decentered towards the inner wall in the same direction as the curvature vectors in cross section 4. Then, the velocity profile in the middle systole is decentered towards the outer wall with increasing flow volume primarily due to the centrifugal force; however, the decentered direction is not always the same as that of the centrifugal force vectors in cross section 4. Instead, it is consistent with that in cross section 3. Because the inertia of the flow increases with the increasing flow volume at the inlet, the effects of the proximal centrifugal force remain. Therefore, the larger change in the torsion may cause the larger decentering eccentricity of the velocity profile.

**Fig. (3):**
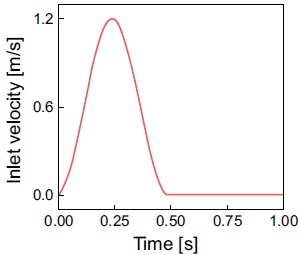
The inlet velocity diagram.

Fig. (**6**) shows the wall shear stress distribution in the middle systole. The qualitative results in the other cardiac cycles are nearly the same. The maximal wall shear stress position is from the ascending aorta to the aortic arch, where the curvature is the largest (Fig. **3**). The circumferential position of the maximal wall shear stress region, however, varies with the torsion of the model (black arrows in models B and C). This is another effect of the aortic torsion on the hemodynamics. The values in Fig. (**6**). indicate the maximal wall shear stress values in the middle systole, which are significantly higher in the post-ASO models B and C.

## DISCUSSION

The geometry of the aorta is assessed *via* its curvature and torsion, and the characteristic changes in these parameters for the post-ASO models are shown in this study. Because the blood flow field and its induced wall shear stress distribution are greatly affected by the geometric shape of the aorta, it is important to consider these characteristic changes for the post-ASO models. A large curvature near the aortic root in a post-ASO model results in an increase in the maximal wall shear stress value in the middle systole. Meanwhile, a large torsion results in a circumferential change in the maximal wall shear stress region. The curvature and torsion are both related to the maximal wall shear stress in the aorta. The effect of the torsion is also explained by the secondary flows. Blood flow in a curved artery with no torsion, for example, has twin symmetric secondary flows due to the centrifugal force. This symmetry is gradually broken with increasing torsion, as shown in the model A. Then, the twin symmetric secondary flows disappear, and one large clockwise flow becomes dominant, as shown in the models B and C. Therefore, the circumferential position of the maximal wall shear stress region will change with increasing torsion. The wall shear stress is well known to play an important role in activating or deactivating various functions of endothelial cells to produce or not produce biochemical substances such as nitric oxide (NO), and this mechanism is also known to be one reason for the initiation and development of vascular pathologies such as atherosclerosis or aortic aneurysms in the long term. Locally high wall shear stress regions are therefore causally related to the localization of the aortic aneurysms [22, 23]. The averaged wall shear stress in the middle systole is nearly the same for both the normal and post-ASO models; however, the maximal wall shear stress in the post-ASO models is significantly higher than that in the normal model. This indicates that the initiation risk for an aortic aneurysm in a post-ASO artery may be higher than that in a normal artery. Furthermore, regarding to the circumferential position of the aneurysm formation, there may be a difference between normal and post-ASO arteries. To reduce the risk of initiating an aneurism, it is suggested that the curvature near the aortic root be decreased during the ASO.

The maximal wall shear stress value was primarily discussed in this paper; however, the change in the wall shear stress in the cardiac cycle is also important. This is because the quantity of biochemical substances produced by the endothelial cells depends on the temporal and spatial gradient as well as the maximal value. The production quantity of prostacyclin (PGI_2_), which is also known to be a vasodilator [24], is strongly dependent on the flow conditions [25]. Steady and pulsatile flows result in physiologically different amounts of PGI_2_ production, even if the time averaged wall shear stress in the cardiac cycle is the same. The effects of temporal differences in the physical stimuli on the production quantities of biochemical substances need to be carefully considered in future studies.

The geometric shape of the artery model is one of the most influential factors in blood flow simulations, especially when a rigid arterial wall is assumed. Therefore, the effect of differences in the selected point numbers from the medical imaging data on determining the computational model was considered. We found that the curvature and torsion of the model did not significantly depend on the number of selected points, if the number of points is 12 or more. The curvature of the model around the ascending aorta, however, changes distinctly in the post-ASO models, and a sufficient number of selected points near the aortic root and through to the aortic arch is necessary for adequate modeling. Next, we investigated the effect of geometric changes in the artery during a cardiac cycle on the flow field because the aorta is rich in elastin and the geometry changes with the pressure wave from the left ventricle. Actually, the medical imaging data from systole and from diastole are slightly different with each other. We constructed some other models from different period in a cardiac cycle, and compared the curvature and torsion among the models. As a result, it was found that these geometric parameters curvature and torsion do not change significantly during a cardiac cycle.

The results in this study are not from a fully fluid and structure coupled simulation but rather from the fluid dynamics in a rigid artery model with a certain cardiac period. The elastic behavior of an arterial wall due to the left ventricle movement, pressure wave propagation [26, 27], and the effect of their interactions on the blood flow field [28] are important for a more realistic and accurate study. Furthermore, the boundary conditions or the existence of branches and tapering are highly influential factors on the local wall shear stress distribution [29]. At the inlet, for example, the aortic valve leaflet affects the velocity profiles around the ascending aorta [11], and the existence of the carotid arteries at the aortic arch [30] affects the blood flow field in that area. A verification of the computational assumptions used in this study is also necessary for future studies.

## CONCLUSION

In summary, we conduct blood flow simulations in the thoracic aorta post-ASO, evaluate geometric changes in the aorta due to the ASO in terms of curvature and torsion, and consider the effect of geometric changes on blood flow in the aorta. A large curvature near the aortic root in a post-ASO model results in an increase in the maximal wall shear stress value in the middle systole. Meanwhile, a large torsion results in a circumferential change in the maximal wall shear stress region. The curvature and torsion are both related to the maximal wall shear stress in the aorta. The maximal wall shear stress in the post-ASO models is significantly higher than that in the normal model. This indicates that the initiation risk for an aortic aneurysm in a post-ASO artery may be higher than that in a normal artery. Furthermore, regarding to the circumferential position of the aneurysm formation, there may be a difference between normal and post-ASO arteries. To reduce the risk of initiating an aneurism, it is suggested that the curvature near the aortic root be decreased during the ASO.

## Figures and Tables

**Fig. (1):**
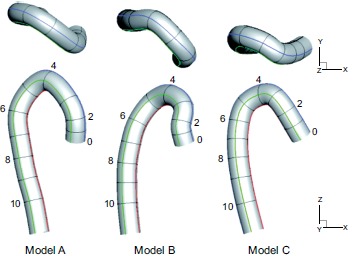
A schematic view of the computational models: the normal model A, and the post-ASO models B and C.

**Fig. (2):**
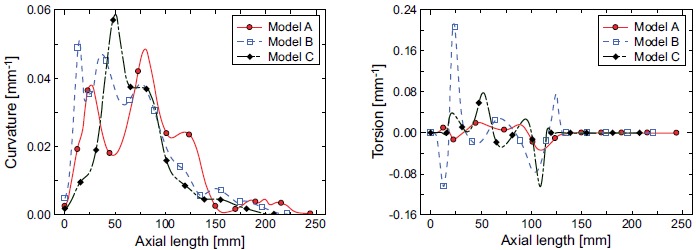
Curvature and torsion diagrams for the three models.

**Fig. (4):**
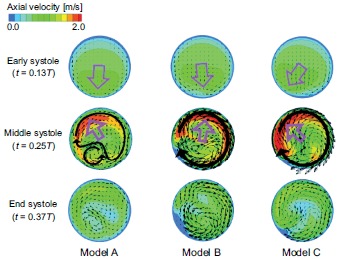
A comparison of the flow fields in cross section 4.

**Fig. (5):**
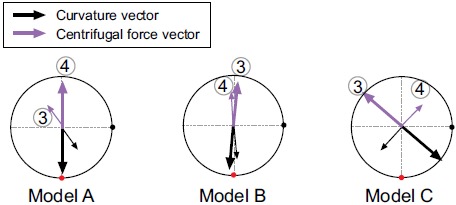
The curvature and centrifugal force vectors at cross sections 3 and 4.

**Fig. (6):**
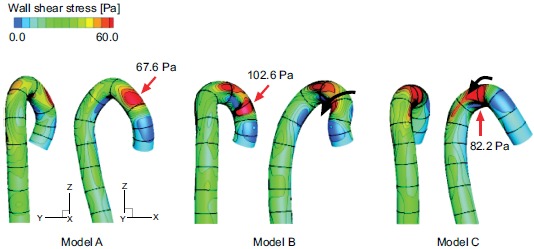
The wall shear stress distribution in the middle systole.

## References

[1] Tare M., Parkington H.C., Coleman H.A., Neild T.O., Dusting G.J. (1990). Hyperpolarization and relaxation of arterial smooth muscle caused
by nitric oxide derived from the endothelium.. Nature.

[2] Tare M., Parkington H.C., Coleman H.A., Neild T.O., Dusting G.J. (1990). Actions of Endothelium-derived Nitric Oxide Include Hyperpolarization of
Vascular Smooth Muscle.

[3] Vorp D.A., Raghavan M.L., Webster M.W. (1998). Mechanical wall stress in abdominal aortic aneurysm: influence of
diameter and asymmetry.. J. Vasc. Surg..

[4] Fillinger M.F., Raghavan M.L., Marra S.P., Cronenwett J.L., Kennedy F.E. (2002). In vivo analysis of mechanical wall stress and abdominal aortic
aneurysm rupture risk.. J. Vasc. Surg..

[5] Shojima M., Oshima M., Takagi K., Torii R., Hayakawa M., Katada K., Morita A., Kirino T. (2004). Magnitude and role of wall shear stress on cerebral aneurysm:
computational fluid dynamic study of 20 middle cerebral artery
aneurysms.. Stroke.

[6] Lasheras J.C. (2007). The biomechanics of arterial aneurysms.. Annu. Rev. Fluid Mech..

[7] Kondo S., Hashimoto N., Kikuchi H., Hazama F., Nagata I., Kataoka H. (1997). Cerebral aneurysms arising at nonbranching sites. An experimental
Study.. Stroke.

[8] Shahcheraghi N., Dwyer H.A., Cheer A.Y., Barakat A.I., Rutaganira T. (2002). Unsteady and three-dimensional simulation of blood flow in the
human aortic arch.. J. Biomech. Eng..

[9] Mori D., Yamaguchi T. (2003). Computational fluid dynamics analysis of the blood flow in the
thoracic aorta on the development of aneurysm. J. Japan. College Angio.

[10] Nakamura M., Wada S., Yamaguchi T. (2005). Computational analysis of blood flow in an integrated model of
the left ventricle and the aorta.. J. Biomech. Eng..

[11] Pasta S., Rinaudo A., Luca A., Pilato M., Scardulla C., Gleason T.G., Vorp D.A. (2013). Difference in hemodynamic and wall stress of ascending thoracic
aortic aneurysms with bicuspid and tricuspid aortic valve.. J. Biomech..

[12] Planché C., Bruniaux J., Lacour-Gayet F., Kachaner J., Binet J.P., Sidi D., Villain E. (1988). Switch operation for transposition of the great arteries in
neonates. A study of 120 patients.. J. Thorac. Cardiovasc. Surg..

[13] Prêtre R., Tamisier D., Bonhoeffer P., Mauriat P., Pouard P., Sidi D., Vouhé P. (2001). Results of the arterial switch operation in neonates with
transposed great arteries.. Lancet.

[14] Williams W.G., McCrindle B.W., Ashburn D.A., Jonas R.A., Mavroudis C., Blackstone E.H. (2003). Outcomes of 829 neonates with complete transposition of the great
arteries 12-17 years after repair.. Eur. J. Cardiothorac. Surg..

[15] McMahon C.J., Ravekes W.J., Smith E.O., Denfield S.W., Pignatelli R.H., Altman C.A., Ayres N.A. (2004). Risk factors for neo-aortic root enlargement and aortic
regurgitation following arterial switch operation.. Pediatr. Cardiol..

[16] Losay J., Touchot A., Capderou A., Piot J.D., Belli E., Planché C., Serraf A. (2006). Aortic valve regurgitation after arterial switch operation for
transposition of the great arteries: incidence, risk factors, and
outcome.. J. Am. Coll. Cardiol..

[17] Asama H., Fukui T., Kimura M., Itoi T., Morinishi K. (2014). Numerical simulation of blood flow and wall shear stress
distribution in the thioracic aorta after arterial switch
operation. Trans. Japan. Soc. Med. Biological Eng..

[18] Chorin A.J. (1997). A numerical method for solving incompressible viscous flow
problems.. J. Comput. Phys..

[19] Tanno I., Morinishi K., Matsuno K., Nishida H. (2006). Validation of virtual flux method for forced convection
flow.. JSME Int. J. Series B.

[20] Morinishi K., Fukui T. (2012). An Eulerian approach for fluid-structure interaction
problems.. Comput. Fluids.

[21] Fukui T., Morinishi K. (2013). Blood flow simulation in the aorta with aortic valves using the
regularized lattice boltzmann method with LES model.. WIT Trans. Built Environ..

[22] Hoi Y., Meng H., Woodward S.H., Bendok B.R., Hanel R.A., Guterman L.R., Hopkins L.N. (2004). Effects of arterial geometry on aneurysm growth:
three-dimensional computational fluid dynamics study.. J. Neurosurg..

[23] Chalouhi N., Hoh B.L., Hasan D. (2013). Review of cerebral aneurysm formation, growth, and
rupture.. Stroke.

[24] Siegel G., Stock G., Schnalke F., Litza B. (1987). Electrical and Mechanical Effects of Prostacyclin in Canine Carotid
Artery.

[25] Frangos J.A., Eskin S.G., McIntire L.V., Ives C.L. (1985). Flow effects on prostacyclin production by cultured human
endothelial cells.. Science.

[26] Fukui T., Parker K.H., Yamaguchi T. (2012). Pulse wave propagation in large blood vessels based on
fluid-solid interactions methods.. Single and Two-phase Flows on Chemical and Biomedical
Engineering.

[27] Fukui T., Parker K.H., Tsubota K., Wada S., Yamaguchi T. (2007). Differentiation of stenosed and aneurysmal arteries by pulse wave
propagation analysis based on a fluid-solid interaction computational
method.. Technol. Health Care.

[28] Fukui T., Parker K.H., Imai Y., Tsubota K-I., Ishikawa T., Wada S., Yamaguchi T. (2007). Effect of wall motion on arterial wall shear
stress.. J. Biomech. Sci. Eng..

[29] Peiffer V., Rowland E.M., Cremers S.G., Weinberg P.D., Sherwin S.J. (2012). Effect of aortic taper on patterns of blood flow and wall shear
stress in rabbits: association with age.. Atherosclerosis.

[30] Niu Y.Y., Chu W.K., Yu H.Y., Wang Y.H. (2004). Numerical prediction of shear stress distribution for a dissected
aorta.. Biomed. Eng. Appl. Basis Commun..

